# Genome-wide survey and expression analysis of F-box genes in chickpea

**DOI:** 10.1186/s12864-015-1293-y

**Published:** 2015-02-13

**Authors:** Shefali Gupta, Vanika Garg, Chandra Kant, Sabhyata Bhatia

**Affiliations:** National Institute of Plant Genome Research, Aruna Asaf Ali Marg, Post Box No. 10531, New Delhi, 110067 India

**Keywords:** Chickpea, F-box genes, Genome-wide, Expression profiles, Stress

## Abstract

**Background:**

The F-box genes constitute one of the largest gene families in plants involved in degradation of cellular proteins. F-box proteins can recognize a wide array of substrates and regulate many important biological processes such as embryogenesis, floral development, plant growth and development, biotic and abiotic stress, hormonal responses and senescence, among others. However, little is known about the F-box genes in the important legume crop, chickpea. The available draft genome sequence of chickpea allowed us to conduct a genome-wide survey of the F-box gene family in chickpea.

**Results:**

A total of 285 F-box genes were identified in chickpea which were classified based on their C-terminal domain structures into 10 subfamilies. Thirteen putative novel motifs were also identified in F-box proteins with no known functional domain at their C-termini. The F-box genes were physically mapped on the 8 chickpea chromosomes and duplication events were investigated which revealed that the F-box gene family expanded largely due to tandem duplications. Phylogenetic analysis classified the chickpea F-box genes into 9 clusters. Also, maximum syntenic relationship was observed with soybean followed by *Medicago truncatula*, *Lotus japonicus* and Arabidopsis. Digital expression analysis of F-box genes in various chickpea tissues as well as under abiotic stress conditions utilizing the available chickpea transcriptome data revealed differential expression patterns with several F-box genes specifically expressing in each tissue, few of which were validated by using quantitative real-time PCR.

**Conclusions:**

The genome-wide analysis of chickpea F-box genes provides new opportunities for characterization of candidate F-box genes and elucidation of their function in growth, development and stress responses for utilization in chickpea improvement.

**Electronic supplementary material:**

The online version of this article (doi:10.1186/s12864-015-1293-y) contains supplementary material, which is available to authorized users.

## Background

The ubiquitin-proteasome pathway is the major regulatory mechanism in a number of cellular processes for selective degradation of proteins and involves three steps: (1) ATP dependent activation of ubiquitin by E1 enzyme (ubiquitin activating enzyme), (2) transfer of activated ubiquitin to E2 (ubiquitin conjugating enzyme) and (3) transfer of ubiquitin to the protein to be degraded by E3 complex (ubiquitin protein ligase). F-box proteins form a subunit of SCF complex (one of the best characterized E3 ligases) and confer specificity for a target substrate to be degraded [1]. The F-box family is among the largest gene family in plants [2] and its size is independent of lineages having no correlation with evolutionary distance, genome size or complexity of the organism [3,4]. Since the discovery of the first F-box protein (Cyclin F) from human [5], numerous F-box proteins have been identified by the presence of a well-conserved N-terminally located 60 amino acids long F-box domain. Although F-box genes are found universally in all prokaryotes and eukaryotes, the number differs greatly from species to species. The number of F-box genes has been observed to be higher in plants than in other systems such as *Drosophila melanogaster* (33 F-box genes) [6] and *Schizosaccharomyces pombe* (18 F-box genes) [7]. Only *Caenorhabditis elegans* has 520 F-box genes, a number comparable to plants [8]. In plants, 694, 687, 337 and 156 F-box genes have been identified in *Arabidopsis thaliana*, *Oryza sativa*, *Populus trichocarpa* and *Vitis vinifera*, respectively [3,9]. Also, Hua et al. [4] identified F-box genes in a number of other plant species for phylogenetic comparisons of F-box proteins. The presence of F-box genes in such large numbers implies that diverse SCF complexes are possible which can recognize a wide array of substrates and have the ability to regulate many important biological processes such as embryogenesis, floral development, plant growth and development, biotic and abiotic stress, hormonal responses and senescence [2]. Therefore, it is of utmost importance to investigate how the F-box gene family evolved in plants. Hence an in-depth analysis of the family can provide a glimpse of the functional divergence, phylogenetics and evolution of the members. However, a great deal of experimental work is required in order to determine the specific biological function of each of these genes comprising the F-box family.

Recently the sequenced and annotated genomes of *kabuli* chickpea [10] and *desi* chickpea [11] were published and therefore it became possible to examine the F-box gene family in chickpea at the whole genome level. With this objective, F-box genes were identified by Hidden Markov Model (HMM)-based search in the *desi* and *kabuli* chickpea genomes and their genomic architecture was established. A phylogenetic tree was constructed to explore the evolutionary forces acting on F-box genes in chickpea. Synteny relationships of the chickpea F-box genes were explored with other legumes such as *Medicago truncatula*, *Lotus japonicus* and soybean along with the non-legume model plant, Arabidopsis. Lastly, digital expression patterns of F-box genes were investigated in various chickpea vegetative tissues as well as in abiotic stress using the transcriptome data publicly available. Besides the evolutionary insights gained by this study, the data also provides a scaffold for future functional analysis of members of this large family of F-box proteins in chickpea.

## Methods

### Identification of F-box genes in chickpea

The Hidden Markov Model (HMM) profiles of F-box (PF00646), F-box-like (PF12937), F-box-like 2 (PF13013), FBA (PF04300), FBA_1 (PF07734), FBA_2 (PF07735), FBA_3 (PF08268) and FBD (PF08387) domains were downloaded from Pfam database [12] and were searched against the annotated proteins in *desi* [13] as well as *kabuli* [14] chickpea genomes (e-value cut-off of 1.0). The redundant sequences were removed and were checked for the presence of F-box domain by SMART [15] and Pfam.

### Sequence analysis

C-terminal domains in F-box proteins were identified using SMART and Pfam with an e-value cut-off of less than 1.0. MEME (Multiple Expectation Maximization for Motif Elicitation) was used to identify the unknown conserved motifs [16] using the following parameters: distribution of motif occurrences: zero or one per sequence, maximum number of motifs: 50 and optimum motif width: ≥ 6 and ≤ 50. The chromosomal locations, length of the coding sequences, gene orientation and exon-intron organization informations were obtained from the chickpea genome webpages [13,14]. WoLF PSORT [17] was used to predict the subcellular localization of proteins. The F-box genes were functionally annotated using Blast2GO [18]. Enrichment analysis was performed using Fisher’s exact test with default parameters (significance threshold of 0.05) available in Blast2GO to identify significantly enriched GO terms. BLASTP search against the *Arabidopsis* peptide sequences [19] was also performed with e-value cut-off of 1e^−10^.

In order to detect splice variants of F-box genes expressing in chickpea, publicly available RNA seq data was used [20]. F-box gene sequences were aligned to *desi* chickpea reference genome [11] by using TopHat 2.0.13 [21] and assembled using Cufflinks [22] to detect isoforms.

### Chromosomal locations and gene duplication analysis

The chromosomal positions of F-box genes provided in the LIS database [14] were utilized for plotting the genes on the eight chickpea chromosomes and visualized using Mapchart [23]. Collinear blocks with e-value ≤ 1e^−10^ were identified by MCSCAN [24] from the Plant Genome Duplication Database [25] and F-box genes falling in these blocks were considered as segmentally duplicated. Genes separated by 10 or fewer genes and >50% similarity at protein level were considered tandemly duplicated [26].

### Synteny analysis

To compare the F-box genes from chickpea with those in other legume species namely, *M. truncatula*, *Glycine max* and *L. japonicus* as well as a non-legume model plant Arabidopsis, BLASTP searches for chickpea F-box genes were conducted using the predicted proteomes of all four species using parameters; e-value ≤ 1e^−10^ and minimum percent identity = 70%. Proteins with unknown chromosomal loci were not used in the analysis. Ideograms were created using Circos [27].

### Phylogenetic analysis

The F-box amino acid sequences were aligned using Bioedit program [28]. A Neighbour-Joining (NJ) phylogenetic tree was constructed using MEGA5 program [29]. Bootstrapping was performed with 1000 replications.

### Digital gene expression analysis

The 454 reads for expression analysis in chickpea tissues- leaf, root, flower bud and pod were retrieved from public repository database, SRA (Sequence Read Archive) available under accession numbers SRX048833, SRX048832, SRX048834 and SRX048835, respectively [30]. For analysis in seed and nodule, the 454 transcriptome data generated in our lab and deposited in SRA under accession numbers SRX125162 and PRJNA214031, respectively, were utilized. For expression analysis of root and shoot under three stress conditions- desiccation, salinity and cold, all Illumina reads were retrieved from SRA database available under accession number SRP034839 [31]. The reads were mapped onto the predicted gene models in *kabuli* [10] and *desi* [11] chickpea genomes using BWA-MEM [32] for 454 reads and BWA [33] for Illumina reads. Mapped reads were extracted using SAM tools [34] and were used for calculating the RPKM (reads per kb per million mapped) values [35]. The RPKM values for F-box genes were utilized for generating the heat maps and k-means clustering using the MeV software [36].

### Quantitative real-time PCR

Root and leaf were harvested from two-week old chickpea seedlings grown under controlled growth conditions. Flowers were tagged on the day of full anthesis and seeds collected at 5 DAA (days after anthesis) and 20 DAA from the field grown chickpea plants. Flower on the day of full anthesis was also collected from the field. Total RNA was isolated from the tissues using LiCl method [37] and cDNA was synthesized from 3 μg of DNase I-treated RNA using M-MLV Reverse transcriptase (Clontech, USA) according to the manufacturer’s instructions. Primer pairs used in quantitative real-time PCR were designed with the Primer Express software (Applied Biosystems, USA) following the manufacturer’s guidelines and have been listed in Additional file 1: Table S1. All the real-time PCR reactions included 2 μl of diluted cDNA, 200 nM of each primer, 2X SYBER GREEN Master Mix (PE-Applied Biosystems), and sterile water for a final volume of 20 μl. The following thermal cycle conditions were used with the ABI 7500 Real Time System (PE Applied Biosystems, USA): (1) incubation at 50°C for 2 m, (2) initial denaturation step of 95°C for 10 m, and (3) 40 cycles of 15 s at 95°C and 1 m at 60°C. CaEF1α (Acc. No. AJ004960) was used as the internal control. All the quantitative real-time PCR experiments were performed twice using two biological replicates and each reaction was run in triplicate. The relative gene expression levels were determined by relative quantification (RQ) method [38].

## Results

### Genome-wide identification and classification of F-box genes in chickpea

The *kabuli* [10] and *desi* [11] chickpea annotated proteins were BLAST searched using HMM profiles of F-box and F-box related domains as queries. Subsequently the sequences were searched for the presence of F-box domain by SMART and Pfam after removing the redundant sequences. A total of 285 potential F-box genes were obtained [see Additional file 2: Table S2]. These comprised of 222 F-box genes from *desi* chickpea and 218 F-box genes from *kabuli* chickpea genome, of which 155 were common.

**Figure 1:**
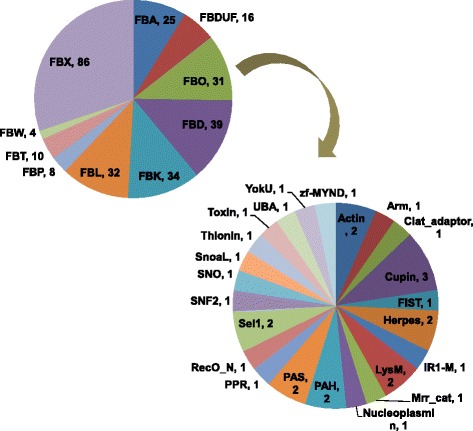
**Classification of chickpea F-box genes.** The number of F-box genes in each group are shown.

Using the SMART and Pfam databases, the C-terminal domains in chickpea F-box genes were identified, based on which, the F-box genes were classified into 10 subfamilies (Figure 1). The most abundant F-box genes (86) were those that did not have any known functional domain other than the F-box domain and were classified as the FBX subfamily. The other 199 genes displayed the presence of one or more known functional domains at their C-terminals and were classified as FBD (39) which contained FBD domain, FBK (34) having kelch repeats, FBL (32) containing LRRs (leucine rich repeats), FBA (25) having F-box associated domain (FBA), FBDUF (16) having domain of unknown function (DUF), FBT (10) containing TUB domain, FBP (8) containing PP2 (phloem protein 2) domain, FBW (4) with WD40 repeats, and FBO (31) containing other domains such as LysM, PAS, PAH, Sel1, Actin, Cupin, PPR, Zf-MyND, SNF2, SNO among others (Figure 1). Further, the unknown motifs in F-box genes of the FBX subfamily, that were found to have no known functional domain other than the F-box, were investigated using MEME. Out of the 50 motifs identified, thirteen were found to be conserved in at least five of the F-box genes [see Additional file 3: Table S3]. Two of these motifs (motifs 3 and 32) were conserved in more than 20 chickpea F-box genes and three motifs (motifs 1, 3 and 4) were found to be statistically significant (e value less than e-100).

**Figure 2:**
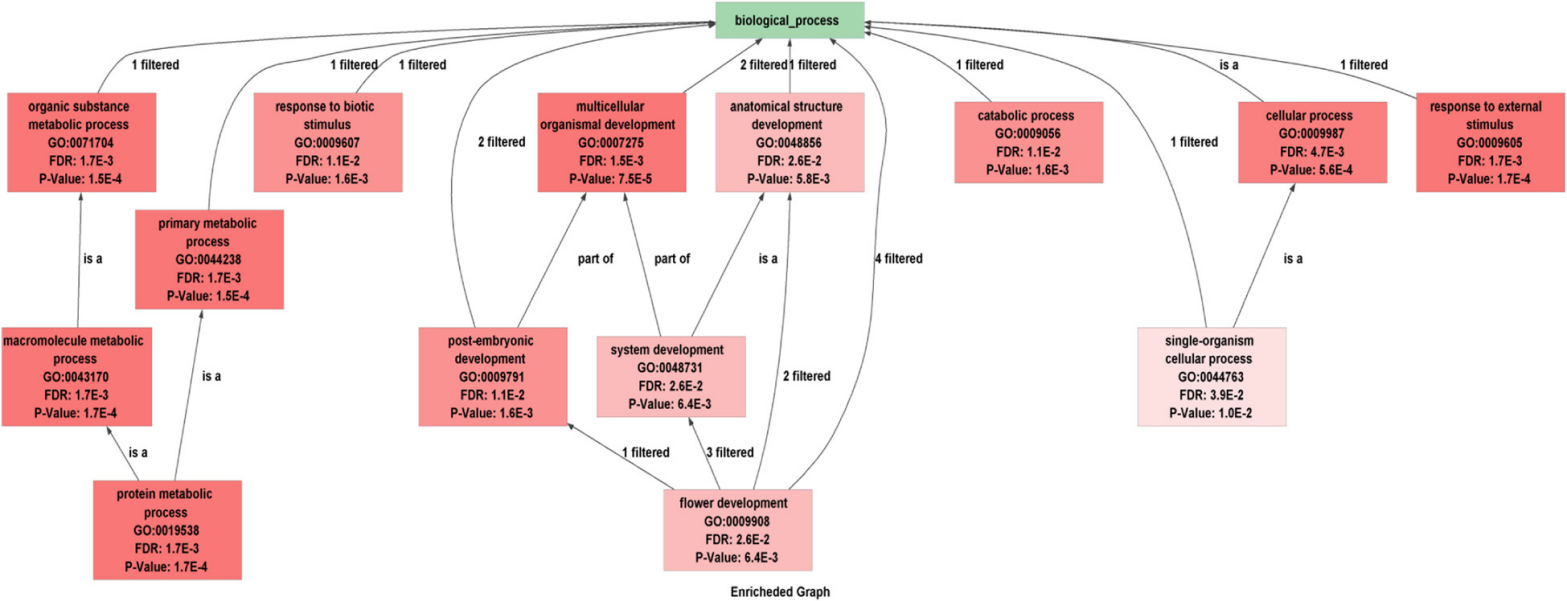
**Gene ontology terms enriched in chickpea F-box genes.** Enrichment of GO terms was determined by Fisher’s exact test.

A Gene Ontology (GO) analysis was also performed using Blast2GO to predict the putative functions of the identified chickpea F-box genes. Most of the F-box genes were predicted to be involved in cellular processes (47) followed by metabolic process (39). Others were found to be involved in essential processes such as response to stimulus, developmental processes, biological regulation, reproduction and signalling. A Fisher’s exact test showed the enrichment of several GO categories such as multicellular organismal development (GO:0007275), primary metabolic process (GO:0044238), response to external stimulus (GO:0009605), protein metabolic process (GO:0019538) and response to biotic stimulus (GO:0009607), followed by catabolic process (GO:0009056) and post-embryonic development (GO:0009791) (Figure 2, Additional file 4: Table S4). Moreover, several homologs of well characterized *Arabidopsis* F-box genes such as FKF1 [39], UFO [40], TIR1 [41], SLOMO [42], AFB [43], among others were observed in chickpea F-box genes sharing 42% to 79% amino acid identity.

### Structural organization of F-box genes and phylogenetic relationships

The gene IDs, length of the coding sequences, protein length and chromosomal locations of all the 285 predicted F-box genes are listed in Additional file 2: Table S2 along with their predicted subcellular locations. The full length coding sequences of the F-box genes ranged from 243 bp (Ca_00042.1) to 4395 bp (Ca_17408.2) with the deduced proteins of 77 to 1363 amino acids. The predicted localization of members of F-box gene family indicated their presence in diverse organelles including cytoplasm, plasma membrane, endoplasmic reticulum, nucleus, mitochondria, chloroplast and extracellular structures. To gain an insight into the structural evolution of the F-box genes in chickpea, their exon-intron organizations were analysed. The number of introns present within each F-box gene ranged from 0 to 16. The F-box genes were classified into four classes depending on their intron composition: intronless, one intron, two introns, three introns and more than three introns per gene. The most abundant class belonged to intronless F-box genes (34.03%; 97) followed by 1 intron (27%; 77), 2 introns (17.5%; 50) and 3 introns (9.1%; 26). Thirty five F-box genes (12.3%) had more than 3 introns. Also, evidence for alternative splicing events occurring in chickpea F-box family was deduced from the splice variants identified for 32 F-box genes [see Additional file 5: Table S5] from *desi* chickpea genome [11]. The number of isoforms ranged from 2 to 4 for each of the 32 F-box genes.

**Figure 3:**
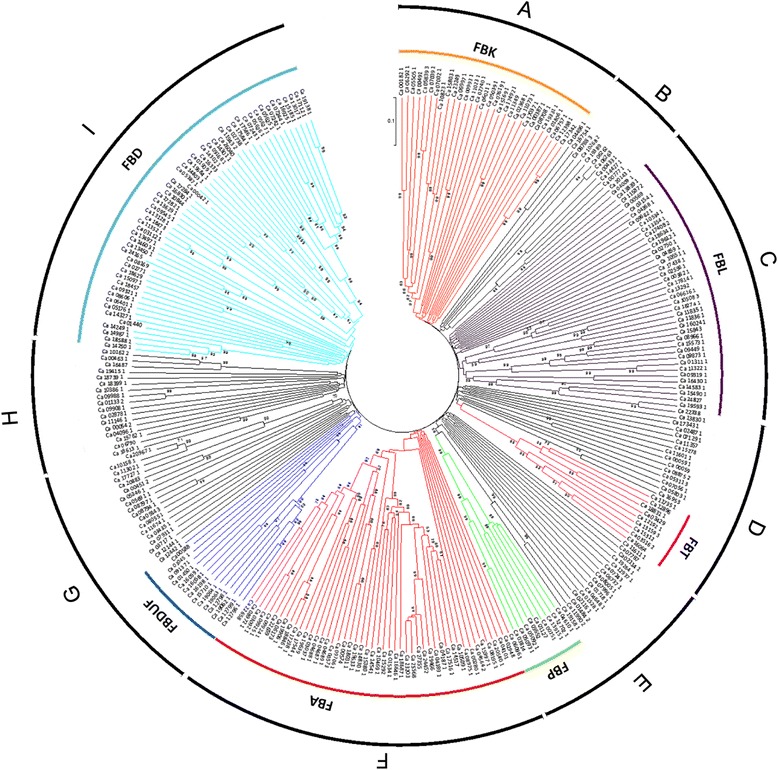
**Phylogenetic analysis of F-box genes in chickpea.** The full-length amino acid sequences were aligned and the Neighbour Joining (NJ) tree was constructed using MEGA5. Bootstrap values (1,000 replicates) are placed at the nodes. Values with >50% supporting the node are indicated. Clades are divided into 9 groups **A-I**. Subfamilies are highlighted by colored segments. Groups B and H contained F-box proteins of FBX subfamily.

A neighbour-joining (NJ) tree was constructed using the full-length protein sequences of all the 285 chickpea F-box genes to study the phylogenetic relationships among them. The phylogenetic tree was divided into 9 clades (Figure 3) in which proteins with the same or similar C-terminal domain organization were found to cluster together. For example, group A mostly contained the members of FBK subfamily. Similarly, all the F-box proteins in group C belonged to FBL subfamily. All the 10 members of FBT subfamily grouped together in group D, a part of a bigger clade. Likewise, 8 members of FBP subfamily were included in group E. Most of the F-box proteins in group F belonged to FBA subfamily. Fifteen of the 17 members belonging to FBDUF subfamily clustered together in a subgroup of clade G. All the members of the FBD subfamily clustered together in group I. Interestingly, proteins with unknown domains either formed their own families (groups B and H), or were scattered within the families formed by proteins with other domains.

### Chromosomal locations and gene duplication events in the chickpea F-box gene family

**Figure 4:**
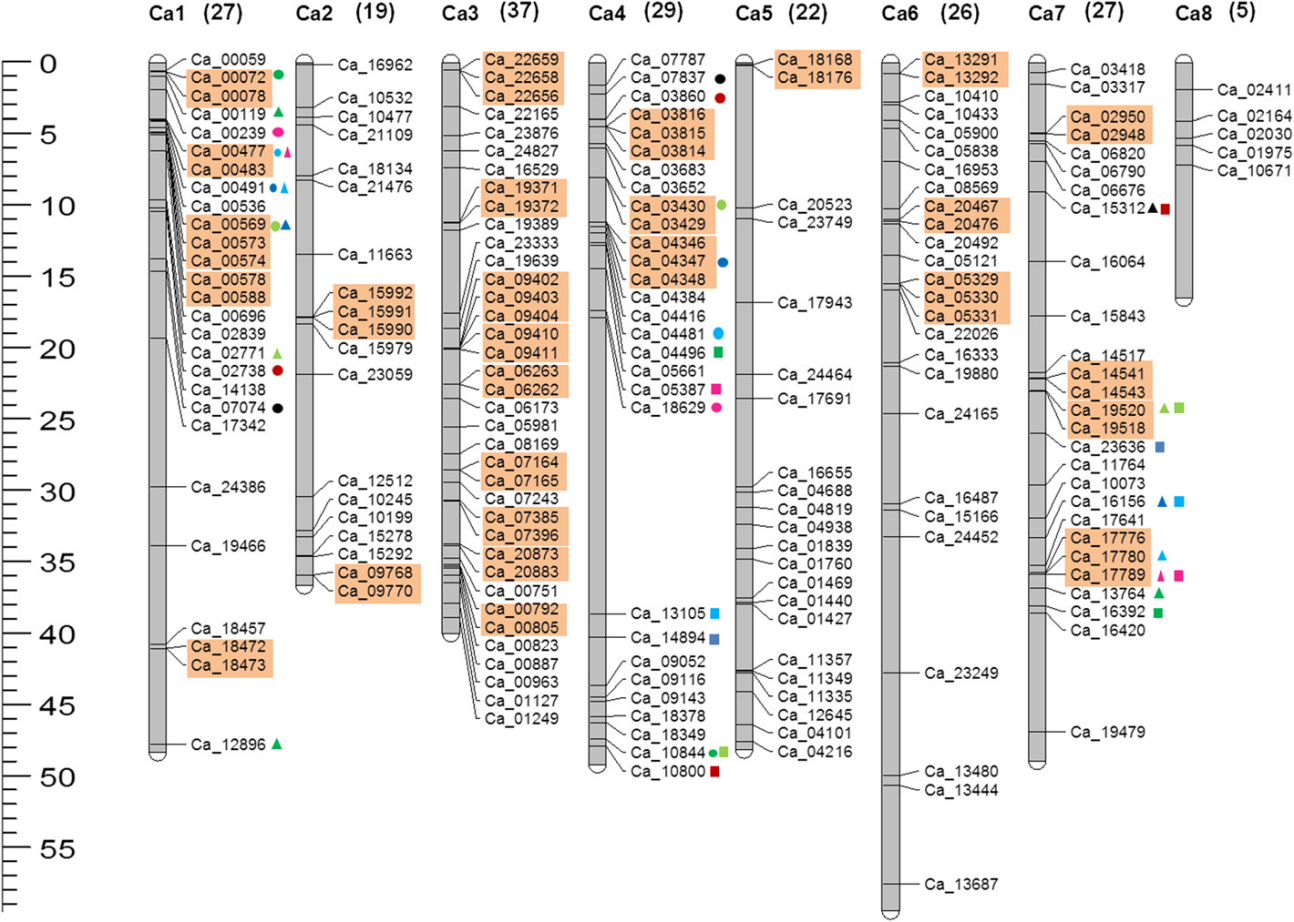
**Chromosomal locations and gene duplication events of F-box genes.** Respective chromosome numbers are indicated at the top of each bar. Numbers in brackets next to each chromosome name represent the number of F-box genes in each chromosome. The scale on the left is in megabases (Mb). Tandemly duplicated genes are shown in orange boxes. Segmental duplications are shown in coloured blocks.

Chromosomal locations of the F-box genes were identified using the draft genome sequences of the *desi* [13] and the *kabuli* genomes [14]. Map positions of 88 F-box protein-encoding genes identified from *desi* chickpea genome could be found, others being present in the scaffolds. Whereas, in case of F-box genes identified from *kabuli* chickpea genome, the chromosomal locations of 192 F-box genes were obtained and hence were considered for mapping of genes on chromosomes as well as for synteny analysis. The F-box genes were found to be almost evenly distributed on all the chromosomes of chickpea except chromosome 8, on which the density of F-box genes was significantly lower (Figure 4). The densities of the genes were relatively higher in specific chromosomal regions, such as the upper arms of chromosome 1, 4 and 6, and the lower arms of chromosome 5 and 7. In contrast, a large middle region on chromosome 4 was devoid of F-box genes. Chromosome 3 was densely populated with F-box genes which had the maximum number of F-box genes (37), followed by 29 genes on chromosome 4.

The contributions made by segmental and tandem duplications in the expansion of the F-box gene family in chickpea were also examined. F-box genes falling within the duplication blocks in the *kabuli* chickpea genome were identified. Among the 192 genes located on chromosomes, 84 (43.75%) arose from duplication events, including 38 gene segmental duplications (13.3%) and 62 gene tandem duplications (21.8%) (Figure 4). The 38 (19 pairs) F-box genes could be assigned to segmental duplication blocks on chromosomes 1, 4 and 7. The 62 tandemly duplicated genes were categorized into 27 groups, 19 of which comprised 2 genes and 8 groups comprised 3 genes. The tandemly duplicated genes were localized on 7 of the 8 chromosomes. Interestingly, several gene clusters expanded through both tandem and segmental duplications, for example, Ca_00072 and Ca_00078, Ca_00477 and Ca_00483 are gene pairs of tandem duplication, and Ca_00072 and Ca_10844, Ca_00477 and Ca_04481 are gene pairs of chromosomal segmental duplication. Moreover, all of the proteins of the duplicated genes had relatively high sequence similarity. For example, Ca_18472 and Ca_18473 from tandem duplication have 88.2% similarity, and Ca_04496 and Ca_16392 from segmental duplication have 70% similarity.

The duplication events within the F-box subfamilies were also analyzed. FBD subfamily was mostly evident in both tandem and segmental duplications. Other subfamilies predominantly involved in tandem duplications were FBX followed by FBA, FBK and FBL. Several F-box genes present in tandem showed retention of their C-terminal domains during duplication events whereas some others showed difference in domains. Segmental duplications contributed more to the expansion of subfamilies FBL and FBX apart from FBD. All but six of the segmentally duplicating pairs belonged to the same subfamily [Additional file 6: Table S6]. Four out of the six pairs had one member belonging to the FBX subfamily.

### Synteny analysis

**Figure 5:**
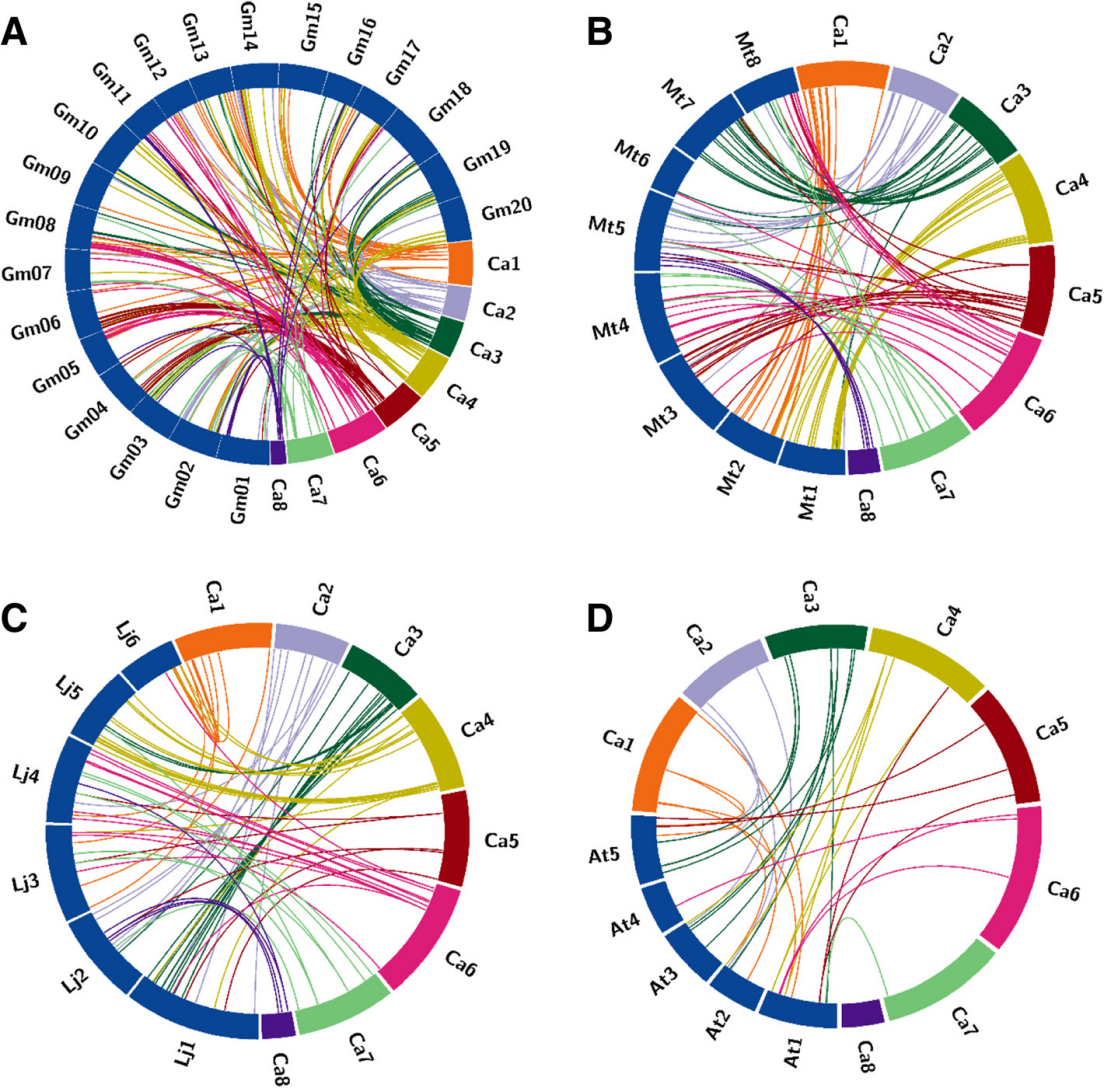
**Synteny analysis of F-box genes.** Synteny between chickpea and **A)** Soybean, **B)** *Medicago truncatula*, **C)** *Lotus japonicus*, and **D)** *Arabidopsis thaliana* is shown. Chickpea chromosomes are depicted as coloured segments whereas others are shown in blue. Colored lines denote syntenic regions between chickpea chromosomes and others.

To explore the evolutionary process of chickpea F-box genes, we analyzed the comparative synteny maps between chickpea and *M. truncatula*, *G. max*, *L. japonicus* and *Arabidopsis* genomes. Amongst the legume species, maximum synteny was found between chickpea and soybean where 106 F-box genes from chickpea shared synteny with 335 F-box genes from soybean (Figure 5A). In contrast 112 chickpea F-box genes were syntenic with 148 F-box genes from *M. truncatula* (Figure 5B). Chickpea and *L. japonicus* were found to have the fewer genes in common with only 94 of the chickpea F-box genes having 127 corresponding orthologs in *L. japonicus* (Figure 5C). On the other hand, only 24 chickpea F-box genes showed synteny with 38 *Arabidopsis* F-box genes (Figure 5D).

### Digital expression analysis of F-box genes in chickpea tissues

**Figure 6:**
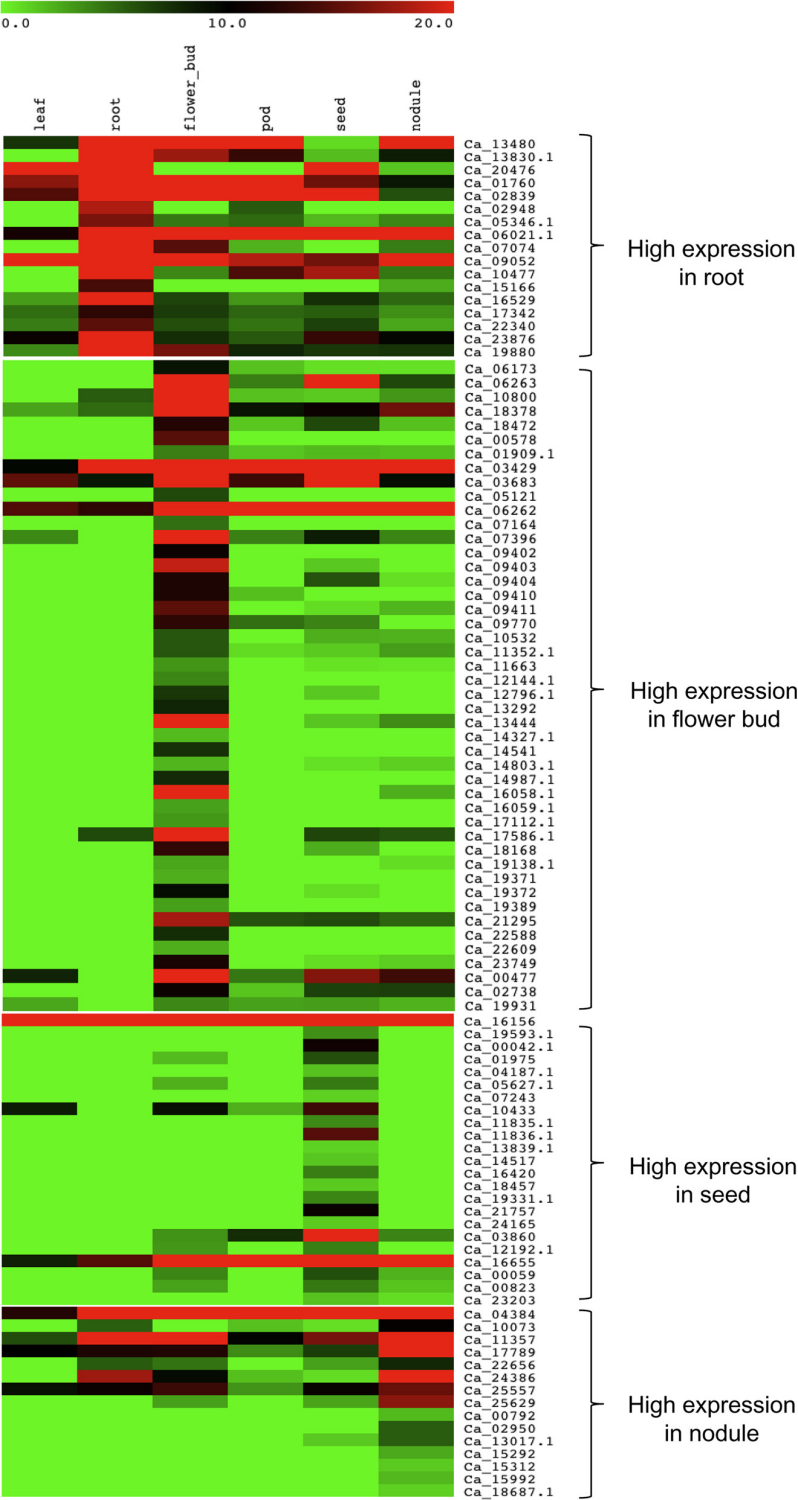
**Heat map showing digital expression profiles of F-box genes in various tissues of chickpea based on RPKM values.** Color key represents RPKM values. Tissue samples are indicated at the top of each lane.

Since tissue specific transcriptomes of chickpea were available for flower bud, pod, leaf, root in SRA database [30] and for seed and nodule, it was possible to investigate the *in-silico* expression profiles of F-box genes in various chickpea tissues. Mapping of the available transcriptome reads revealed expression patterns of 265 F-box genes out of 285 which were retrieved in terms of RPKM values. Out of 265 genes, 258 were found to have RPKM ≥1.0 in at least one of the tissues and were considered as expressed genes [see Additional file 7: Table S7A]. Hierarchical clustering of the expression profiles showed that several F-box genes exhibited preferential expression in one or more of the chickpea tissues. Moreover, tissue specific F-box genes could also be identified. Analysis using k-means clustering resulted in identification of several clusters of which 4 major clusters with genes showing high expression in different tissues are represented in Figure 6. The maximum number of F-box genes (46) were found to have high expression in flower bud followed by 23 in seed, 16 in root and 15 in nodule [see Additional file 8: Table S8A]. Moreover F-box genes having tissue specific expression varied from 15 in flower bud, 13 in seed, 6 in nodule, 2 in leaf and 1 in pod. Several chickpea F-box genes showing tissue specific expression profiles exhibited high similarity with well documented F-box genes in *Arabidopsis*. For example, Ca_05121 sharing 62.88% homology with UFO [40] was observed to be flower bud specific. Moreover other F-box genes exhibiting high expression levels in flower bud included genes such as Ca_07787 that showed homology with FBL17 [44] (60.7%) and Ca_10410 with FKF1 [39] (78.68%). Ca_10433 which showed predominant transcript accumulation in seed was distantly related to MEE11 [45] F-box gene (42.86% homology). Ca_16962 chickpea F-box gene which shared 73.88% amino acid identity with ARABIDILLO1 [46] F-box gene of *Arabidopsis* accumulated preferentially in root.

**Figure 7:**
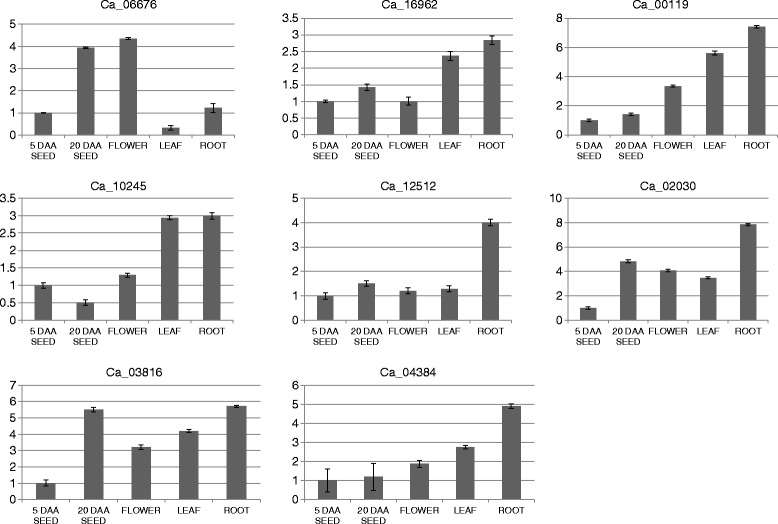
**Relative expression levels of F-box genes in different chickpea tissues.** Total RNA was extracted from seed, flower, leaf and root. For each gene, the relative expression levels were obtained by normalization with chickpea EF1α. The error bars indicate standard deviations.

To validate the expression patterns, several candidate genes were selected for quantitative RT-PCR (Figure 7). The transcript accumulation patterns were analysed in root, leaf, flower and seed of chickpea. The results were fairly similar to the RPKM data. For example, Ca_12512 showed preferential expression in root. Ca_06676 showed predominant transcript accumulation in seed and flower while Ca_02030 expressed ubiquitously in all the tissues with comparatively less expression in leaf.

### Digital expression analysis of F-box genes under abiotic stress

**Figure 8:**
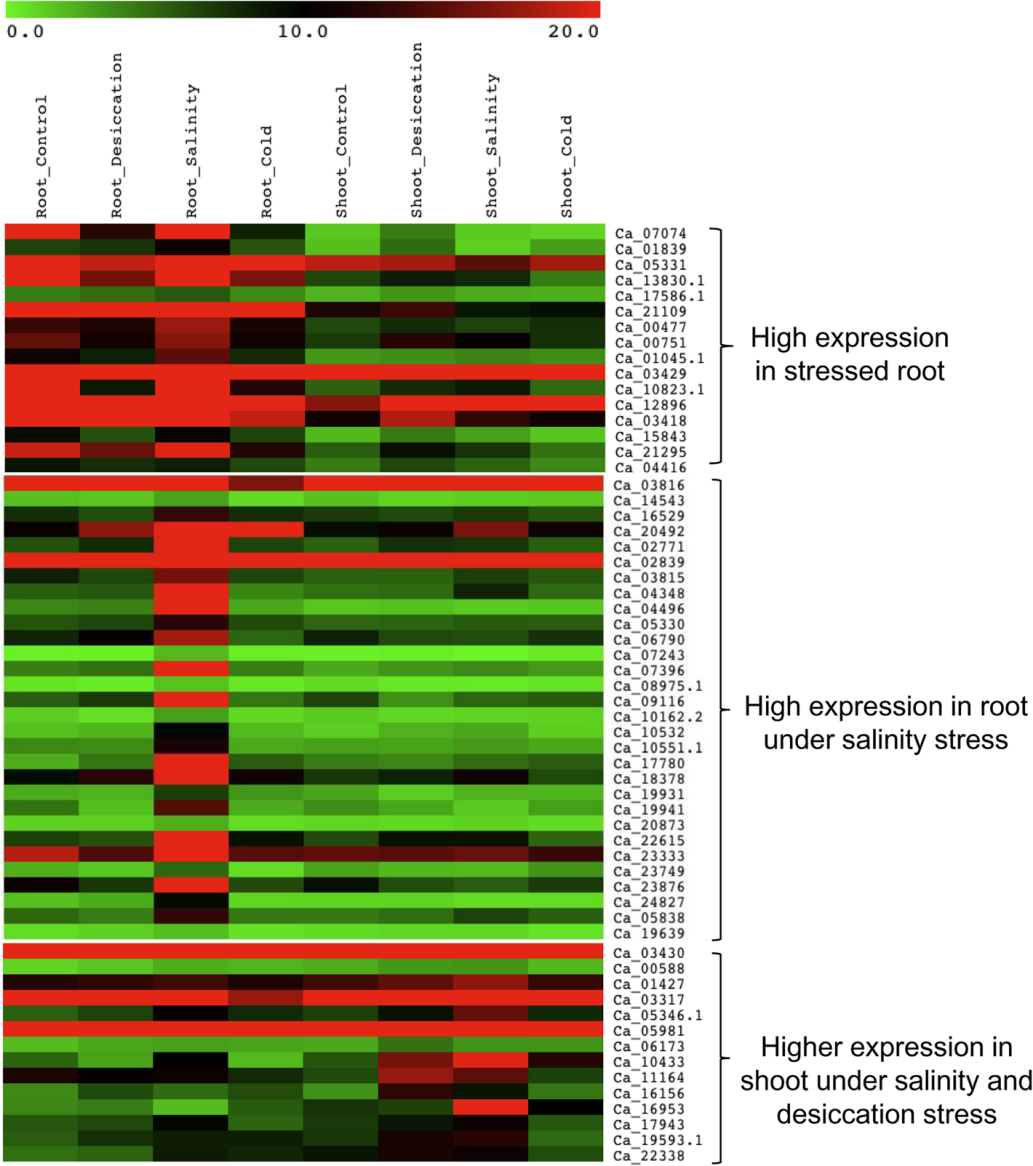
**Heat map showing digital expression profiles of F-box genes (based on RPKM values) in chickpea root and shoot under stress.** The three abiotic stress conditions were- desiccation, salinity and cold. Color key represents RPKM values. Tissue samples are indicated at the top of each lane.

Chickpea F-box genes were also analysed for their *in-silico* expression profiles using the available root and shoot transcriptomes of chickpea under three abiotic stress conditions- desiccation, salinity and cold [31]. Out of 265 genes for which RPKM values could be calculated, 220 were found to have RPKM values ≥ 1.0 in at least one tissue [see Additional file 7: Table S7B]. Many of 220 F-box genes, for which RPKM values could be retrieved, exhibited differential transcript accumulation in one or more of the stress conditions. The k-means clustering resulted in clusters showing genes expressing high in one or few stresses. For example 16 genes were found to be expressing at high levels in root tissue under various stress conditions as compared to shoot. Whereas 30 genes showed high expression in root under salinity stress. Moreover 14 genes showed high expression in shoot under desiccation and salinity stress (Figure 8; Additional file 8: Table S8B).

## Discussion

The ubiquitin/ proteasome pathway is the major regulatory mechanism for selective protein degradation in a wide variety of cellular processes [2]. Plants contain the largest known number of F-box proteins suggesting the need for F-box proteins throughout the plant life cycle. The fact that they play critical roles in many aspects of plant growth and development, make F-box proteins a very important subject for studies. It will be quite attractive to develop improved chickpea varieties through transgenic approaches by over/ under expressing the target F-box gene leading to selective protein degradation and hence altering the outcome of the cellular process involved. Such altered expression of F-box proteins have been implicated recently in plants such as Arabidopsis to confer salinity tolerance [47], in tobacco to regulate primary carbohydrate metabolism [48] and to enhance the polyphenol production and UV tolerance in Arabidopsis [49].

The F-box superfamily has previously been phylogenetically and evolutionarily characterized in various plant species [4,50-52]. However, a comprehensive analysis of the F-box gene family in chickpea was lacking but became possible with the recent availability of chickpea genome sequence [10,11]. Thus, 285 F-box genes were identified from the complete chickpea genome. Comparison of the number of F-box genes in chickpea with those in other plants [50,52,4] revealed that chickpea had less number of F-box genes than *Arabidopsis* (694), rice (687) and legumes such as *G. max* (702) and *M. truncatula* (1148). The number of F-box genes have been reported to be species specific [4] and not proportional to the sizes of the genomes [3]. Moreover Hua et al. [4] have attributed the large variation in the F-box gene numbers across different plants to extensive gains/losses of F-box genes. Since the chickpea genome has been a result of a number of gene loss and duplication events [10], this may have led to the underrepresentation of F-box genes in chickpea. Moreover, the relatively fewer F-box genes in chickpea indicate that F-box proteins in chickpea may have acquired the function to recognize multiple substrates or there might be prevalence of alternative pathways for protein degradation in chickpea.

Domain analysis of the chickpea F-box genes revealed that a large fraction (30.17%) of the predicted genes did not have any other known functional domain other than the F-box. However analysis of the remaining (≃70%) F-box genes revealed the presence of several domains such as LRR, kelch repeats, FBD, FBA, WD40, PP2, PAS/PAH, TUB and PPR at their C-termini, allowing their classification into 10 groups. Most F-box genes have been shown to contain different protein-protein interaction domains at their C-termini which are known to interact with various substrates [50,52]. Similarly in other species also F-box genes with unknown or no C-terminal domains were most abundant as also observed in *Arabidopsis* [50], rice [52] and *M. truncatula* [4] [Additional file 9: Table S9]. However, amongst the C-terminal domain containing F-box genes, the FBD type which is thought to be associated with nuclear processes [53] was the most abundant in chickpea in contrast to DUF domain containing F-box genes in rice [52], FBA domain containing F-box genes in *M. truncatula* [4] and LRR repeats containing F-box genes in *Arabidopsis* [50]. The proportion of FBA domain containing F-box genes was similar in chickpea and *M. truncatula* [4] and was much higher in comparison to rice [52]. The FBA domain containing F-box genes have been shown to be related to pollen recognition in *Arabidopsis* [54]. The FBT subfamily consisting of TUB domain (first detected in mouse to be involved in controlling obesity [55]) consisted of 10 members in chickpea as was also observed in *Arabidopsis* [50] whereas the rice and *M. truncatula* FBT subfamilies comprised of 14 members [52] and 7 members [4], respectively. The FBP subfamily comprised of eight PP2 domain containing F-box genes. Eighteen lectin-related domain containing F-box genes were identified in the genome-wide survey of F-box genes from *Arabidopsis* [50]. However, this domain could not be identified in many other plants studied [4,52]. It has been suggested [56] that few phloem lectins (Phloem protein 2), typically associated with phloem function, have acquired F-box domains during their evolution and may have diverged from their phloem function in order to interact with glycoproteins to bring about protein degradation. WD40 repeat containing F-box genes were lowest in number as also observed in rice [52] and *Arabidopsis* [50]. This indicated that the C-terminal domains determine specific protein-protein interactions in important biological processes and critically define the function of the F-box gene. Additionally, thirteen new motifs could be predicted by MEME which may be important for protein-protein interactions. However, the functional significance of these motifs needs to be validated experimentally. Moreover F-box proteins have been shown to be involved in diverse biological processes [2]. The GO annotations of the F-box genes carried out in our study also confirmed this suggesting their probable involvement in essential biological pathways. Functional characterization of most of the F-box genes till date has been done in the model plant, *Arabidopsis* and there homologs were found to occur in chickpea also where they may be performing similar functions. For example, close homologs of TIR1 [41] (Ca_03430; 79.42% protein identity), AFB5 [43] (Ca_23059; 68.13%) and SLOMO [42] (Ca_09143; 63.49%) which are known to be involved in plant growth and development through auxin homeostasis could be identified in the chickpea F-box genes.

An examination of the exon-intron organization of the F-box genes demonstrated the prevalence of 34% intronless genes in the family which is a distinct feature of the F-box genes as has also been observed in *Arabidopsis*, rice and *Populus* [9]. Also, it was observed that most members of a subfamily had similar intron/exon structures suggesting close structural relationships between the F-box genes within a subfamily in chickpea. Moreover, the chickpea F-box genes sharing high homology with *Arabidopsis* F-box genes showed similar exon/intron organizations. Further, to obtain an overall picture of the evolutionary relationship of chickpea F-box proteins, a phylogenetic tree was constructed, which divided the family into 9 clades. The organization of F-box proteins in the phylogenetic tree suggests that F-box genes with similar C-terminal domains coevolved just as observed in *Arabidopsis* [50] and rice [52]. The fact that members of each clade usually have identical domain organization suggested that they function to interact with the same or similar substrates. The location of proteins with unknown domains implied the complexity of their evolutionary lineage. Moreover, the similar phylogenetic tree topologies of chickpea, *Arabidopsis* [50] and rice [52] suggest a common evolutionary lineage for this gene family in plant species from dicots and monocots.

Gene duplication is thought to be an important means of gene family expansion and functional diversity during evolution, which may occur through chromosomal segmental duplication or tandem duplication [57]. Previous reports have indicated that duplication events have contributed to the amplification of F-box gene family [4]. Moreover, whole genome sequencing of chickpea established that about 69% of predicted chickpea genes have a history of duplication after the divergence of the legumes from *A. thaliana* and grape [10]. It is possible that F-box genes expanded in such large numbers to regulate proteolysis of proteins arising out of duplicated genes. Our analysis of gene duplication events within the chickpea F-box family revealed that 84 of 192 (43.7%) F-box genes were duplicated genes, 38 genes (13.3%) had arisen out of segmental duplication and 62 (21.8%) genes were a result of tandem duplication indicating that tandem duplications contributed more to the expansion of the F-box gene family in chickpea than segmental duplication. Similar results were observed in rice [9,52] and *Arabidopsis* [9,50] thereby indicating that duplication of F-box genes in plant genomes may have utilized a common mechanism. When analyzing the duplication events occurring at the subfamily level, it was observed that the F-box gene subfamilies in chickpea showed a bias towards the mode of duplication for their expansion. Most of the genes involved in tandem and segmental duplications belonged to the FBD, FBX and FBL subfamilies. This could have resulted due to an increased rate of duplication events within these subfamilies in chickpea. According to a recent study by Navarro-Quezada et al. [58], the F-box subfamilies expand in waves depending on the mode as well as the timing of duplication events. It was also suggested that the F-box protein subfamilies possibly share a common evolutionary pattern which generally involves massive duplication and rapid gene birth/death during the course of evolution. Also, the expansion in the subfamilies seems to be species-specific as could be observed on comparing the F-box subfamilies of chickpea, Arabidopsis, rice and *M. truncatula* [Additional file 9: Table S9].

Apart from this, four out of the six segmentally duplicated pairs had one member belonging to the FBX subfamily suggesting the diversification of the C-terminal domains during the course of evolution. Several tandemly duplicated gene pairs belonging to different subfamilies further supported the possibility of diversification of F-box genes. The F-box domain and C-terminal domains are reported to be showing strong tendency of negative and positive selection, respectively through the course of evolution leading to the sequence diversification of C–terminal domains and conservation of F-box domain [58]. This may also be the reason for the dramatic variation between the lengths of F-box proteins as has also been observed in other plant species [4] which may have led to the gain or loss of amino acids within an F-box protein for adaptive evolution to recognize different substrates.

Sequence comparison of related genes across species from different taxa and within the genome makes it possible to reconstruct the evolutionary history of a gene family [59]. The highly variable number of F-box genes observed in closely related legumes i.e. chickpea, *M. truncatula* and soybean, stimulated us to explore the syntenic relationships amongst the legumes as well as the non-legume model plant *Arabidopsis*. The largest synteny was observed with soybean probably because among legumes, soybean has the largest number of syntenic blocks due to its recent polyploid ancestry [10]. Similar level of orthology shared between chickpea and other legumes (37% of chickpea F-box genes with soybean; 39% with *M. truncatula* and 33% with *L. japonicus*) supports their close evolutionary relationships. Also, gene loss and gene duplication events were evident within the different species analyzed in this study.

Transcriptomes serve as a useful resource for preliminary gene expression analysis [60] which may also be useful for predicting putative functions. The transcript abundance analysis based on RPKM values revealed that most of the F-box genes expressed preferentially and sometimes specifically in one or more of the chickpea tissues which was validated experimentally by selecting several candidate F-box genes for real-time PCR analysis. Further, several of the chickpea F-box genes found expressing preferentially in the tissue specific clusters correlated well with their homologs reported from other plants. F-box genes such as UFO [40], DOUBLE TOP [61], DDF1 [62] and FKF1 [39] have been shown to have a role in floral development. Homologs of UFO (Ca_05121) and FKF1 (Ca_10410) were observed to be expressing preferentially in the flower bud tissue in chickpea indicating their putative participation in floral development. Also, Ca_07787, a homolog of the FBL17 F-box gene of *Arabidopsis* (60.7% protein identity) involved in pollen development [44], had higher RPKM values in flower bud as well as in nodule tissue of chickpea. On the other hand, F-box genes such as MEE11 [45], MAX2 [63] and ORE9 [64] have been reported to have roles in embryo development, seed dormancy and leaf senescence, respectively. It will be interesting to investigate the function of their homologs such as Ca_10433 which was homologous to MEE11 F-box gene and expressed specifically in chickpea seed tissue. Several F-box genes such as KUK [65], VFB [66], ARABIDILLO [46] and MAIF1 [67] have been shown to be involved in functions related to root development. Based on high homology with ARABIDILLO and preferential expression of Ca_16962 in root, it could be suggested that it may also have a similar role in promoting lateral root development in chickpea. Therefore it could be inferred that F-box genes expressing in a tissue specific manner most likely participated in important functions specific to the tissue type whereas the ubiquitously expressed F-box genes were involved in general cellular machinery.

An attempt was also made to analyse the digital expression profiles of the chickpea F-box genes under three abiotic stress conditions- desiccation, salinity and cold by utilizing the already available transcriptome data [31]. It was seen that several F-box genes specifically expressed in abundance under different abiotic stress conditions in concordance with previous reports in rice [52] and other species [68]. The roles of several F-box genes such as MAX2 [69], FBP7 [70], DOR [71] and MAIF1 [67] have been well established during abiotic stress conditions. Their chickpea homolog such as Ca_19880 (60% homology with MAX2) exhibited comparatively higher expression during salinity stress in root [69] thereby indicating a putatively similar role in chickpea. Overall, these findings indicate that the F-box genes might be mediating specific responses to various stress conditions such as desiccation, salinity and cold.

## Conclusions

A comprehensive genome-wide analysis of F-box gene family was carried out for the first time in an important legume crop i.e. chickpea which led to the identification and classification of 285 F-box genes. The structural and phylogenetic analysis helped in identifying conserved F-box subfamilies present in the chickpea genome. Expansion of the chickpea F-box gene family occurred largely through tandem duplications was also established. Synteny analysis with *M. truncatula*, soybean, *L. japonicus* and *Arabidopsis* revealed evolutionary insights. Most significantly the digital expression profiles of the F-box genes across different tissues as well as under three abiotic stress conditions helped in identifying several putative genes specifically involved in varied physiological and molecular processes occurring in chickpea tissues during development and stress. This study would serve as a foundation for selection and characterization of candidate genes to be used for improvement of crop chickpea.

### Availability of supporting data

The accession numbers of the datasets used in the digital expression analysis of this article are included within the article and can be retrieved from public repository database, SRA (http://www.ncbi.nlm.nih.gov/sra/).

## Additional files

Additional file 1: Table S1. Primers used in the study. Additional file 2: Table S2. F-box genes in chickpea. Information including domain present, ORF length, protein length, and number of introns within ORF, genomic locus (chromosomal location), gene orientation and predicted subcellular localization of the protein is provided for each F-box gene. Additional file 3: Table S3. Putative motifs predicted in F-box proteins of FBX family by MEME. Additional file 4: Table S4. Enriched GO terms associated with F-box genes in chickpea. Additional file 5: Table S5. List of isoforms identified for F-box genes in *desi* chickpea genome. Additional file 6: Table S6: F-box genes present on duplicated chromosomal segments of chickpea. Additional file 7: Table S7A. RPKM values of F-box genes in different chickpea tissues. **Table S7B.** RPKM values of F-box genes under three abiotic stress conditions. Additional file 8: Table S8A. k-means clusters representing higher expression of some F-box genes in different chickpea tissues. **Table S8B.** k-means clusters representing higher expression of some F-box genes under three abiotic stress conditions. Additional file 9: Table S9. Sizes of common subfamilies in four plant species, Arabidopsis, rice, *M. truncatula* and chickpea.
