# Supplementary material for: Genome-wide survey and expression analysis of F-box genes in chickpea
Source: BMC Genomics. 2015 Feb 13;16(1):67. doi: 10.1186/s12864-015-1293-y (PMC4340835; doi:10.1186/s12864-015-1293-y)
Supplement: Additional file 1: Table S1. — Primers used in the study. [file 12864_2015_1293_MOESM1_ESM.pdf]

Table S1. List of primers used in the study

Primer Name	Primer sequence
Ca_16962	F: GCTGAAGCAGTTATGCGAGATG
	R: GGCCACGTTGGCATTCAC
Ca_00119	F: CCCTGAAGATGAAGCGAAAAA
	R: TCTGATACTTGAACCCAACATGCT
Ca_06676	F: CAGAGGAAGCAACTCAGAACGA
	R: GAGAAGGACAGCCATGAGCAA
Ca_10245	F: GGAATGCCCCATCTGTTTGA
	R: GGACCATCATGACCCCAAAG
Ca_12512	F: GGAACCAGGCTTGCTTTTGTC
	R: GGAAGAATTGGATTTCACAACTGA
Ca_02030	F: CGGAGGGTAGAAGAGACTGAGACT
	R: GATTTACACACGGAAGCACAAGAA
Ca_03816	F: GACGAAGGCTAAGATACCAGATTGT
	R: CTTCCTCAGAACGAGTTCGATTG
Ca_04384	F: TGCGGCTCCTACGACAACA
	R: AATAATCTTGTCATGGTCAGACTGTGT
