# Supplementary material for: Genome-wide survey and expression analysis of F-box genes in chickpea
Source: BMC Genomics. 2015 Feb 13;16(1):67. doi: 10.1186/s12864-015-1293-y (PMC4340835; doi:10.1186/s12864-015-1293-y)
Supplement: Additional file 3: Table S3. — Putative motifs predicted in F-box proteins of FBX family by MEME. [file 12864_2015_1293_MOESM3_ESM.pdf]

Table S3 Putative motifs predicted in F-box proteins of FBX family by MEME.

Motif No.	Length of motif	No. of sites	Sequence	Sequence logo	E-value
1	50	9	P[DV]MP[TI][LIV][GS]VL[GM]DCLCFS[YH][YDS][PF][NK][KG][TFI][NEH]FV[IL]W[KQ]M[QK][EI][FY]GV[EQY]ESW[TV][QK][FL][LI]KIS[YH][QH][NT][LQ][HQ]	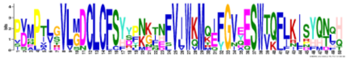	4.8e-188
3	21	26	SLL[RQ]L[RK]CV[CS]KSWR[AS]LIS[DN]PxF	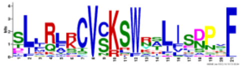	5.1e-152
4	29	8	V[ED]QF[RV]IVSLDL[GS]TETY[NR]Q[YL][LM]LPRGFD[EK][MV]P	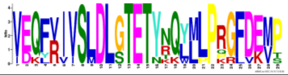	8.6e-103
5	21	10	V[RK][IV][FLH][TS][LF]G[DT]N[VS]W[RK][DNR]I[EQ][SD]FP[VT][VG][PV]	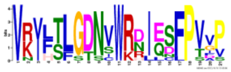	3.7e-069
6	17	17	xF[AS]FGYD[NH]STDTYKVVA	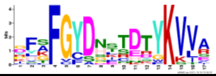	1.2e-066
7	41	5	[FN][EG]DTLIL[RTA][SR][HSN][QK][GRE]Q[SV]I[FLR]YN[WK][RI][DNH]N[ITR][VL][EY][EK][IT][NR][VI]T[IN][GNTV][NI][NY][RTW][TY][TL][DA][YN][VQ][EF]	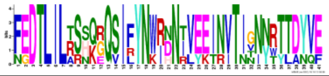	9.1e-062
10	28	9	[ATV]VRT[SC][VI]LS[KT]RWI[DH][LV]W[KT][LN]I[TFP][HN]L[DHS]FD[DW]S[FH][MN]	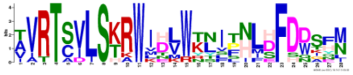	4.2e-059
13	29	9	[FR][KD][NES][DGRS]G[VK][YFH][LV][GNS]GT[VIL]NWLA[MIS][DKT][ND][DY][FT][PES]Y[SW]V[LI][KV][NS][HL]	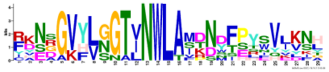	1.5e-048
14	28	7	[NK][EG][SW][AI][ST][FV][FI][FL][LE][NR][PR]V[TQ][KN][AI][RH]I[MQ][IY][PA][SN]KL[FY][FI]PSK	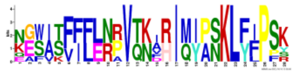	1.4e-047
25	29	5	[GHN]F[CQS][YA][NH][KVI][KL][TL]RQ[LI][TH][KE][IV][ED][CRG][IL][KPA][LEIM][GL][FNY][FY][KE][VA][WL][LR][HQ][TA]N	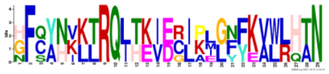	1.5e-026
32	22	21	x[DS]LARA[SA]C[VT]C[KS]x[WL]Nx[AL]AS[DS][DEP]xL	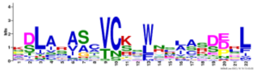	3.9e-020
40	29	5	K[VF][VF][KQT][FMV][KQV][GR]F[NTA][GA][KNT][EKP][HLC][ED][LV][CNS][FL][VA][KQ][FHLY][LVM][MI][KAT][NKR]A[YALT][MTA][LM][EK]	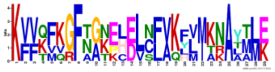	1.2e-015
48	21	6	L[AN][KR][DC][ER][AN]S[GR][EN]L[YE][LI][IL]Y[MR]E[CG][MN]V[EQ]Y	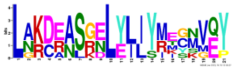	7.8e-014
