# Supplementary material for: Genome-wide survey and expression analysis of F-box genes in chickpea
Source: BMC Genomics. 2015 Feb 13;16(1):67. doi: 10.1186/s12864-015-1293-y (PMC4340835; doi:10.1186/s12864-015-1293-y)
Supplement: Additional file 4: Table S4. — Enriched GO terms associated with F-box genes in chickpea. [file 12864_2015_1293_MOESM4_ESM.pdf]

Table S4 Enriched GO terms associated with F-box genes in chickpea

GO ID	GO Term	FDR	P-Value
GO:0007275	multicellular organismal development	1.51E-03	7.49E-05
GO:0044707	single-multicellular organism process	1.51E-03	7.49E-05
GO:0044767	single-organism developmental process	1.51E-03	7.49E-05
GO:0032502	developmental process	1.51E-03	7.49E-05
GO:0032501	multicellular organismal process	1.51E-03	7.49E-05
GO:0044699	single-organism process	1.51E-03	8.30E-05
GO:0044238	primary metabolic process	1.68E-03	1.55E-04
GO:0071704	organic substance metabolic process	1.68E-03	1.55E-04
GO:0009605	response to external stimulus	1.68E-03	1.70E-04
GO:0043170	macromolecule metabolic process	1.68E-03	1.70E-04
GO:0019538	protein metabolic process	1.68E-03	1.70E-04
GO:0008152	metabolic process	1.70E-03	1.87E-04
GO:0009987	cellular process	4.70E-03	5.61E-04
GO:0009607	response to biotic stimulus	1.11E-02	1.58E-03
GO:0009056	catabolic process	1.11E-02	1.58E-03
GO:0009791	post-embryonic development	1.11E-02	1.63E-03
GO:0050896	response to stimulus	1.84E-02	2.88E-03
GO:0048856	anatomical structure development	2.59E-02	5.84E-03
GO:0048731	system development	2.59E-02	6.43E-03
GO:0009908	flower development	2.59E-02	6.43E-03
GO:0061458	reproductive system development	2.59E-02	6.43E-03
GO:0003006	developmental process involved in reproduction	2.59E-02	6.43E-03
GO:0044702	single organism reproductive process	2.59E-02	6.43E-03
GO:0090567	reproductive shoot system development	2.59E-02	6.43E-03
GO:0048608	reproductive structure development	2.59E-02	6.43E-03
GO:0048367	shoot system development	2.59E-02	6.43E-03
GO:0022414	reproductive process	2.59E-02	6.43E-03
GO:0044763	single-organism cellular process	3.94E-02	1.01E-02
GO:0003824	catalytic activity	4.76E-02	1.27E-02
