# Supplementary material for: Genome-wide survey and expression analysis of F-box genes in chickpea
Source: BMC Genomics. 2015 Feb 13;16(1):67. doi: 10.1186/s12864-015-1293-y (PMC4340835; doi:10.1186/s12864-015-1293-y)
Supplement: Additional file 6: Table S6: — F-box genes present on duplicated chromosomal segments of chickpea. [file 12864_2015_1293_MOESM6_ESM.pdf]

Table S6 F-box genes present on duplicated chromosomal segments of chickpea.

Gene ID	Subfamily	Chr. No.	Gene ID	Subfamily	Chr. No.
Ca_00072	FBX	1	Ca_10844	FBD	4
Ca_00239	FBX	1	Ca_18629	FBX	4
Ca_00477	FBL	1	Ca_04481	FBX	4
Ca_00491	FBK	1	Ca_04347	FBX	4
Ca_00569	FBL	1	Ca_03430	FBL	4
Ca_02738	FBD	1	Ca_03860	FBD	4
Ca_07074	FBX	1	Ca_07837	FBX	4
Ca_00119	FBK	1	Ca_13764	FBK	7
Ca_00477	FBL	1	Ca_17789	FBL	7
Ca_00491	FBK	1	Ca_17780	FBO	7
Ca_00569	FBL	1	Ca_16156	FBL	7
Ca_02771	FBDUF	1	Ca_19520	FBD	7
Ca_12896	FBT	1	Ca_15312	FBT	7
Ca_04496	FBP	4	Ca_16392	FBP	7
Ca_05387	FBX	4	Ca_17789	FBL	7
Ca_13105	FBL	4	Ca_16156	FBL	7
Ca_14894	FBA	4	Ca_23636	FBA	7
Ca_10844	FBD	4	Ca_19520	FBD	7
Ca_10800	FBT	4	Ca_15312	FBT	7

Six segmental duplication pairs belonging to different subfamilies are shown in red.
