# Supplementary material for: Genome-wide survey and expression analysis of F-box genes in chickpea
Source: BMC Genomics. 2015 Feb 13;16(1):67. doi: 10.1186/s12864-015-1293-y (PMC4340835; doi:10.1186/s12864-015-1293-y)
Supplement: Additional file 9: Table S9. — Sizes of common subfamilies in four plant species, Arabidopsis, rice, M. truncatula and chickpea. [file 12864_2015_1293_MOESM9_ESM.pdf]

Table S9 Sizes of common subfamilies in four plant species, Arabidopsis, rice, *M. truncatula* and chickpea

**Species (Total no. of F-box genes)**	**FBX**	**FBA**	**FBK**	**FBDUF**	**FBD**	**FBL**	**FBT**	**FBW**
Arabidopsis[1] (694)	374 (53.8%)	-	97 (13.9%)	-	-	160 (23%)	10 (1.4%)	2 (0.2%)
Rice[2] (687)	465 (67.6%)	4 (0.5%)	25 (3.7%)	66 (9.6%)	17 (2.4%)	61 (8.8%)	14 (2%)	2 (0.2%)
M. truncatula[3] (1148)	334 (29%)	84 (7%)	25 (2%)	13 (1%)	27 (2%)	27 (2%)	7 (1%)	-
Chickpea (285)	86 (30%)	25 (9%)	34 (12%)	16 (6%)	39 (14%)	32 (11%)	10 (4%)	4 (1.4%)

[1]Data taken from Gagne et al. (2002)
[2] Data taken from Jain et al. (2007)
[3]Data taken from Hua et al. (2011)
C-terminal domains found common in the four species have been shown.
